# First Quantitative Imaging of Organic Fluorine within Angiogenic Tissues by Particle Induced Gamma-Ray Emission (PIGE) Analysis: First PIGE Organic Fluorine Imaging

**DOI:** 10.3390/pharmaceutics3010088

**Published:** 2011-03-09

**Authors:** Sébastien Lavielle, Karine Gionnet, Richard Ortega, Guillaume Devès, Victor Kilarski, Katia Wehbe, Andreas Bikfalvi, Gérard Déléris

**Affiliations:** 1 Université Bordeaux Segalen, CNRS, UMR 5084, 146 rue Leo Saignat, 33076 Bordeaux, France; 2 Université Bordeaux 1, CNRS, UMR 5084, Chemin du Solarium, 33175 Gradignan, France; 3 Université Bordeaux 1, INSERM U920, Avenue des Facultés, 33405 Talence, France

**Keywords:** ion beam imaging, fluorine imaging, cancer angiogenesis, tracers peptide synthesis

## Abstract

PET (Positron Emission Tomography) allows imaging of the *in vivo* distribution of biochemical compounds labeled with a radioactive tracer, mainly 18F-FDG (2-deoxy-2-[18F] fluoro-D-glucose). 18F only allows a relatively poor spatial resolution (2-3 mm) which does not allow imaging of small tumors or specific small size tissues, e.g. vasculature. Unfortunately, angiogenesis is a key process in various physiologic and pathologic processes and is, for instance, involved in modern anticancer approaches. Thus ability to visualize angiogenesis could allow early diagnosis and help to monitor the response of cancer to specific chemotherapies. Therefore, indirect analytical techniques are required to assess the localization of fluorinated compounds at a micrometric scale. Multimodality imaging approaches could provide accurate information on the metabolic activity of the target tissue.

In this article, PIGE method (Particle Induced Gamma-ray Emission) was used to determine fluorinated tracers by the nuclear reaction of 19F(p,p′γ)19F in tissues. The feasibility of this approach was assessed on polyfluorinated model glucose compounds and novel peptide-based tracer designed for angiogenesis imaging. Our results describe the first mapping of the biodistribution of fluorinated compounds in both vascularized normal tissue and tumor tissue.

## 1. Introduction

Angiogenesis is a process by which new capillaries sprout from pre-existing blood vessels [1]. It is involved both in physiological and pathological processes. Physiological angiogenesis occurs in the female reproductive system [2] as during wound healing [3]. In contrast, pathological neo-angiogenesis occurs in a number of diseases, such as cancer or rheumatoid arthritis [4]. Imaging of angiogenesis is of great interest not only for tumors but other angiogenesis-related diseases, such as cerebral infarct, both allowing earlier diagnosis and therapeutic follow-up. Current technology does not allow both resolutive and sensitive molecular imaging due to lack of specific targeting to vessels.

Molecular imaging is the noninvasive visualization of physiological as well as pathological cellular processes at a molecular level. It is used to provide characterization and *in vivo* measurement of biological processes in living animals and humans. Positron emission tomography (PET), which uses labeled molecular probes is currently considered to be the leading technology for imaging [5]. It is a non-invasive and highly sensitive technique which offers three-dimensional imaging of radiolabeled tracers in tissues. A PET-tracer emits a positron which, shortly after emission, combines with an electron, where after an annihilation takes place and two 511 keV photons are emitted in opposite directions. The resulting photons are then detected by a PET scanner. This technique has two main advantages. It is highly sensitive and, as it is based on biochemical behavior of tracer, it allows functional follow-up of tumors, as well as the effect of therapies. However, its main pitfall is its poor spatial resolution typically of 2–3 mm [6-8] which is not sufficient to allow the detection of small tumors or metastases.

In an attempt to validate molecular PET targets at the cell scale level, and afterwards to transfer previously obtained information when performing PET scan at the millimetric level, we inferred that the use of a multiscale imaging method, beginning with imaging stable fluorine-19 tracers at a cell scale, could be used for further interpretation of PET results at a millimetric level. Among methods allowing elemental detection with high sensitivity and high spatial resolution, ion beam analysis and associated imaging appeared to be a suitable method. Ion beam analyses are based on interactions, at both the atomic and the nuclear level, between accelerated charged particles and the bombarded material. These interactions lead to the emission of particles and multiple radiations. Energies generated by the latter are characteristic of the elements of the sample material. Ion beam analysis also allows the quantitative imaging of the distributions of chemical elements at a micrometric scale. Spatial resolutions are compatible with the analysis of molecular interactions between specific tracers and their targets.

We have very recently described for the first time that the detection of fluorine-19 within diluted organic samples could be performed by PIGE analysis (Particle Induced Gamma-ray Emission) using the nuclear reaction 19F(p,p′γ)19F [9]. To date, PIGE is mainly used on geological [10], environmental [11] and mineralized biological materials [12]. As for the latter, most studies have been conducted on teeth, but none was performed in biological tissues.

We were able, for the first time, to determine and quantify the localization of fluorinated tracers in vascularized tissues. We first performed a study using highly concentrated solutions of two analogs of glucose (FDG and a polyfluorinated derivative) to assess the feasibility of this approach. In a second step, we developed a new and specific angiogenesis fluorinated tracer, called F13-CBO-P11 (F13-cVEGI), to study its biodistribution at a micrometric scale.

## 2. Experimental Section

### 2.1. General methods

All chemicals were purchased from Sigma-Aldrich and Alfa-Aesar. Preloaded resin and amino acids were purchased from Novabiochem.

Reactions were followed by thin layer chromatography (TLC) using precoated Baker silica gel plates 60F_254_ (Merck) using various eluent gradients and spots were visualized by dipping the plate in a 1% KMnO_4_ aqueous solution followed by heating.

Peptide synthesis was performed on an Applied Biosystems 433A automated peptide synthesizer by Fmoc/t-Bu batch solid-phase synthesis (Applied Biosystems).

One-dimensional ^1^H, ^13^C NMR, two-dimensional gradient selected heteronuclear multiple bond correlation (HMBC) and gradient selected heteronuclear single quantum correlation (HSQC) spectra were recorded on a Bruker Avance 300 spectrometer (300 MHz for ^1^H and 75 MHz for ^13^C). Chemical shifts are reported in d values (ppm), by reference to the hydrogenated residues of deuterated solvent as internal standard. Signals are described as s, d, t and m for singlet, doublet, triplet and multiplet respectively.

Infrared (IR) analysis was performed on a Spotlight 300 FT-IR spectral imaging system equipped with a Spectrum One spectrometer (Perkin-Elmer, France).

Mass spectra were run using a matrix-assisted laser desorption ionization-time of flight (MALDI-TOF) Reflex III (Bruker).

As described in our previous methodologic publication [9], ion beam experiments were carried out by using 3.4 MeV proton beam (focused to 5 μm) delivered by the HVEE in-line 3.5 MeV Singletron accelerator at the Centre d'Etudes Nucléaires de Bordeaux Gradignan (CENBG) on AIFIRA (Applications Interdisciplinaires des Faisceaux d'Ions en Région Aquitaine) platform. Data treatments, chemical map reconstruction and definition of region of interest were made using Supavisio software. Local concentrations of chemical elements and depth profiles of RBS spectra were performed by SimNRA program [13]. PyMCA software was used to fit the peaks of fluorine on PIGE spectra [14].

### 2.2. Synthesis of probes and precursors

#### 3-allyl-1,2:5,6-di-O-isopropylidene-α-D-glucofuranose **(2)**

Sodium hydride (350 mg, 11.5 mmol) was added portion-wise to a solution of 1,2:5,6-di-O-isopropylidene-α-D-glucofuranose **(1)** (2 g, 7.7 mmol) in toluene. The reaction was heated at 110 °C for 2 h. Allyl bromide (1.35 mL, 15.4 mmol) was added and the reaction was stirred at 110 °C for 18 h. 10 mL water was added and the aqueous phase was extracted with diethyl ether (2 × 25 mL). The combined organic layers were dried over MgSO_4_ and solvent was removed under vacuum to yield yellowish oil that was purified by chromatography (75:25 n-hexane/ethyl acetate). Compound **2** (2.16 g, 94%) was obtained as a colorless oil. R_f_ (hexane:ethylacetate 3:1) = 0.65. MS (m/z): calcd for C_15_H_24_O_6_ 300.35 found 300.16. ^1^H NMR (CDCl_3_) δ ppm: 1.27–1.46 (s, 12H, CH3), 3.79–3.86 (m, 2H, H-6a, H-6b), 3.94 (m, 1H, H-3), 4.14– 4.21 (m, 4H, H-4, H-5 and C*H_2_*CH=CH_2_), 4.51 (m, 1H, H-2), 5.14–5.28 (m, 2H, CH_2_CH=C*H_2_*); 5.84– 5.91 (m, 2H, H-1 and CH_2_C*H*=CH_2_). ^13^C NMR (CDCl_3_) δ ppm: 25.8–27.5 (4C, CH3), 65.0 (C-3), 67.9 (C-6), 70.1 (*C*H_2_CH=CH_2_), 72.6 (C-4), 77.9 (C-5), 80.5 (C-2), 105.6 (C-1), 108.9 and 111.7 (2C, C(CH_3_)_2_), 116.6 (CH_2_CH=*C*H_2_); 134.3 (CH_2_*C*H=CH_2_).

#### 3-(4,4,5,5,6,6,7,7,8,8,9,9,9-tridecafluoro-2-iodo-nonyloxy)-1,2:5,6-di-O-isopropylidene-α-D-glucofuranose **(3)**

To a cooled (0 °C) and stirred solution of 1.33 mmol of the compound **2** in 6 mL acetonitrile and 3 mL water, 360 mg (4.28 mmol) of sodium bicarbonate were added under argon. Then 765 μL of 1-iodoperfluorohexane (3.3 mmol) and 420 mg of sodium dithionite (2.4 mmol) were added and the mixture was allowed to warm slowly to room temperature. After 1.5 h, 100 mL of diethyl ether were poured into the reaction mixture. The resulting solution was washed with brine and water (50 mL of each), before drying over MgSO_4_. After filtration the solution was evaporated to dryness under reduced pressure. The resulting crude product was used without further purification for the hydrodeiodination step. R_f_ (hexane:ethylacetate 3:1) = 0.63. MS (m/z): calcd for C_21_H_24_F_13_IO_6_ 746.30 found 746.04. ^1^H NMR (CDCl_3_) δ ppm: 1.27–1.46 (s, 12H, CH3), 2.54–2.74 (m, 2H, CHIC*H_2_*CF_2_), 3.03–3.09 (m, 1H, CHI), 3.79–3.86 (m, 2H, H-6a, H-6b), 3.90 (m, 2H, OC*H_2_*CHI), 3.94 (m, 1H, H-3), 4.14–4.21 (m, 2H, H-4, H-5), 4.51 (m, 1H, H-2), 5.84 (d, 1H, H-1). ^13^C NMR (CDCl_3_) δ ppm: 15.6 (CHI), 25.8–27.5 (4C, CH3), 37.6 (CHI*C*H_2_CF_2_), 46.2 (O*C*H_2_CHI), 65.0 (C-3), 67.9 (C-6), 72.6 (C-4), 77.9 (C-5), 80.5 (C-2), 105.6 (C-1), 108.9 and 111.7 (2C, C(CH_2_)), 106.7–120.3 (CF_2_ and CF_3_).

#### 3-(4,4,5,5,6,6,7,7,8,8,9,9,9-tridecafluoro-nonyloxy)-1,2:5,6-di-O-isopropylidene-α-D-glucofuranose **(4)**

Compound **3** (2.4 g, 3.2 mmol) was dissolved in a mixture of 30 mL ethanol and 30 mL ethyl acetate. After addition of 1 mL of triethylamine and 150 mg palladium on charcoal (10%), the iodine was cleaved off in a hydrogen atmosphere (1 atm) overnight. After removal of the charcoal by filtration, the solution was concentrated and the resulting product purified via column chromatography (75:25 n-hexane/ethyl acetate). Compound **4** (1.26 g, 63%) was obtained as a yellow oil. R_f_ (dichloromethane) = 0.36. MS (m/z): calcd for C_21_H_25_F_13_O_6_ 620.40 found 620.14. ^1^H NMR (CDCl_3_) δ ppm: 1.27–1.46 (s, 12H, CH3), 1.84–1.87 (m, 2H, OCH_2_C*H_2_*), 2.11–2.26 (m, 2H, CH_2_C*H_2_*CF_2_), 3.55– 3.70 (m, 2H, OC*H_2_*CH_2_), 3.79–3.86 (m, 2H, H-6a, H-6b), 3.94 (m, 1H, H-3), 4.14–4.21 (m, 2H, H-4, H-5), 4.51 (m, 1H, H-2), 5.84 (d, 1H, H-1). ^13^C NMR (CDCl_3_) δ ppm: 21.0 (OCH_2_*C*H_2_), 25.8–27.5 (4C, CH3), 28.1 (CH_2_*C*H_2_CF_2_), 66.1 (C-6), 69.1 (O*C*H_2_CH_2_), 70.0 (C-3), 72.6 (C-4), 77.9 (C-5), 80.5 (C-2), 105.6 (C-1), 108.9 and 111.7 (2C, C(CH_3_)_2_), 106.7-120.3 (CF_2_ and CF_3_).

#### 3-(4,4,5,5,6,6,7,7,8,8,9,9,9-tridecafluoro-nonyloxy)-α-D-glucose **(5)**

Compound **4** (800 mg, 1.29 mmol) was added at room temperature in 35 mL of trifluoroacetic acid and 35 mL of distilled water. The reaction was stirred for 3 h. The solvent was then evaporated off and remaining residues were codistilled with ethanol and toluene (3 times each). The resulting crude product was recrystallized from ethyl acetate. Compound **5** (596 mg, 86%) was obtained as a white powder. R_f_ (hexane:ethylacetate 1:1) = 0.13. MS (m/z): calcd for C_15_H_17_F_13_O_6_ 540.27 found 540.08. ^1^H NMR (CDCl_3_) δ ppm: 1.63–1.68 (m, 2H, OCH_2_C*H_2_*), 2.08–2.13 (m, 2H, CH_2_C*H_2_*CF_2_), 3.11 (m, 1H, H-3), 3.35–3.46 (m, 2H, OC*H_2_*CH_2_), 3.59–3.66 (m, 2H, H-6a, H-6b), 3.73–3.76 (m, 2H, H-4, H-5), 3.81 (m, 1H, H-2), 4.26 (d, 1H, H-1). ^13^C NMR (CDCl_3_) δ ppm: 22.5 (OCH_2_*C*H_2_), 29.1 (CH_2_*C*H_2_CF_2_), 62.9 (C-6), 71.5 (C-4), 72.6 (O*C*H_2_CH_2_), 75.4 (C-5), 78.1 (C-3), 86.7 (C-2), 98.4 (C-1), 106.7–120.3 (CF_2_ and CF_3_).

#### 1-Allyloxy-8-Hydroxy-3,6-Dioxaoctane **(7)**

Sodium hydride (3 g, 0.08 mol) was added portion-wise to a solution of triethyleneglycol (20 mL, 0.15 mol) in dry THF (50 mL). The solution was stirred at room temperature for 15 min. Then allyl bromide (4.3 mL, 0.05 mol) was added and stirring was maintained at room temperature for 1 h. Then solvent was evaporated under reduced pressure and the residue was dissolved in dichloromethane. The solution was filtrated and washed with brine (40 mL). The aqueous phase was extracted with dichloromethane (2 × 100 mL). The organic phase was dried over MgSO_4_ and solvent were evaporated under reduced pressure. The resulting yellowish oil was purified by chromatography (n-hexane/ethyl acetate 70:30 and then 50:50). Compound **7** (6.93 g, 73%) was obtained as a colorless oil. R_f_ (hexane:ethylacetate 1:1) = 0.15. MS (m/z): calcd for C_9_H_18_O_4_ 190.24 found 190.12. ^1^H NMR (CDCl_3_) δ ppm: 3.53–3.76 (m, 12H); 3.99 (d, 2H, C*H_2_*CH=CH_2_, ^3^*J*_H-H_ = 5.61 Hz); 5.23–5.24 (m, 2H, CH=C*H_2_*); 5.84–5.94 (m, 1H, C*H*=CH_2_). ^13^C NMR (CDCl_3_) δ ppm: 61.6 (CH_2_OH); 70.2–70.5 (CH_2_O); 72.2 (*C*H_2_CH=CH_2_); 72.5 (*C*H_2_CH_2_OH); 117.1 (CH=*C*H_2_); 134.6 (*C*H=CH_2_).

#### 1-[(p-toluenesulphonyl)oxy-]-9-Allyl-3,6,9-Trioxaoctane **(8)**

A solution of compound **7** (2.14 g, 11.3 mmol) in distilled triethylamine (30 mL) was cooled to 0 °C. Then tosyl chloride was added (3.22 g, 16.9 mmol) to the medium and was stirred at room temperature for 24 h. Then the reaction mixture was poured into ice and ethyl acetate was added (100 mL). The aqueous phase was extracted with ethyl acetate (2 × 100 mL). The combined organic layers were washed with a 2% (v/v) acetic acid aqueous solution and H_2_O (50 mL each), and then dried over MgSO_4_. Solvent was removed under vacuum to yield yellowish oil that was purified by chromatography (75:25 n-hexane/ethyl acetate). Compound **8** (3.1 g, 80%) was obtained as a colorless oil. R_f_ (hexane:ethylacetate 1:1) = 0.64. MS (m/z): calcd for C_16_H_24_O_6_S 344.42 found 344.13. ^1^H NMR (CDCl_3_) δ ppm: 2.44 (s, 3H, CH_3_); 3.52–3.66 (m, 10H, CH_2_CH_2_O); 4.01 (d, 2H, C*H_2_*CH=CH_2_, ^3^*J*_H-H_ = 5.61 Hz); 4.14 (t, 2H, CH_2_OTs, ^3^*J*_H-H_ = 4.7 Hz); 5.14–5.28 (m, 2H, CH=C*H_2_*); 5.84–5.94 (m, 1H, C*H*=CH_2_); 7.33 (d, 2H, CH_Ar_, ^3^*J*_H-H_ = 8.1 Hz); 7.77 (d, 2H, CH_Ar_, ^3^J_H-H_ = 8.1 Hz). ^13^C NMR (CDCl_3_) δ ppm: 21.2 (CH3); 68.2–68.9 (CH_2_CH_2_O); 70.1–70.2 (*C*H_2_CH=CH_2_, *C*H_2_CH_2_OTs); 71.7 (CH_2_OTs); 116.6 (CH=*C*H_2_); 127.5–129.5 (CH_Ar_); 132.5 (C_Ar_SO_2_); 134.3 (*C*H=CH_2_); 144.5 (C_Ar_CH_3_).

#### N-2-(2-[2-(2-allyloxy-ethoxy)-ethoxy]-ethyl)-phtalimide **(9)**

To a solution of compound **8** (3.1 g, 9 mmol) in distilled DMF (100 mL) was added potassium phthalimidate (1.83 g, 9.9 mmol). The solution was heated at 120 °C for 12 h and subsequently cooled to room temperature. Solvent was evaporated under reduced pressure and the resulting residue was dissolved in CH_2_Cl_2_ (100 mL). H_2_O (100 mL) was added. The organic phase was extracted with CH_2_Cl_2_ (3 × 100 mL) and dried over MgSO_4_. Filtration and evaporation of the solvent yielded to compound **9** (2.73 g, 94%). R_f_ (hexane:ethylacetate 3:1) = 0.58. MS (m/z): calcd for C_17_H_21_NO_5_ 319.35 found 319.14. ^1^H NMR (CDCl_3_) δ ppm: 3.34–3.47 (m, 10H, CH_2_CH_2_O); 3.52 (t, 2H, CH_2_NPht, ^3^*J*_H-H_ = 5.76 Hz); 3.67 (t, 2H, C*H_2_*CH_2_NPht, ^3^*J*_H-H_ = 5.85 Hz); 3.77 (d, 2H, C*H_2_*CH=CH_2_, ^3^*J*_H-H_ = 5.61 Hz); 4.91–5.09 (m, 1H, CH=C*H_2_*); 5.57–5.76 (m, 2H, C*H*=CH_2_); 7.48–7.55 (m, 2H, CH_Ar_); 7.57–7.63 (m, 2H, CH_Ar_). ^13^C NMR (CDCl_3_) δ ppm: 37.1 (CH_2_NPht); 67.8 (*C*H_2_CH_2_NPht); 69.3–72.0 (CH_2_CH_2_O); 116.9 (CH=*C*H_2_); 123.1 (CH_Ar_); 132.0 (C_Ar_); 133.8 (CH_Ar_); 134.6 (*C*H=CH_2_); 168.1 (C=O). IR (cm^−1^): 2906; 2867; 1774; 1713; 1615; 1107; 1006; 928.

#### N-2-(2-(2-[2-(4,4,5,5,6,6,7,7,8,8,9,9,9-tridecafluoro-nonyloxy)-ethoxy]-ethoxy)-ethyl)-phtalimide **(10)**

5.6 g of compound **9** (17.5 mmol) and sodium bicarbonate (4.7 g, 56 mmol) were added under argon in 100 mL of a cooled solution (0 °C) 2:1 acetonitrile/water. Then 1-iodoperfluoroalkane (6 mL, 24.8 mmol) and sodium dithionite (5.5 g, 31.6 mmol) were added to the medium. The reaction was stirred at room temperature for 3 h. After completion of the reaction (tlc control) 300 mL of diethyl ether were poured onto the reaction mixture. The resulting solution was washed with brine and water (150 mL of each), before drying over MgSO_4_. After filtration the solution was evaporated to dryness under reduced pressure. The resulting crude product (9.6 g) was used without further purification for the hydrodeiodination step.

This latter was dissolved in ethanol (100 mL), 2 mL of triethylamine and 300 mg palladium on charcoal (10%) were added under a hydrogen atmosphere (1 atm) overnight. After filtration, the solvent was evaporated under reduced pressure and the resulting residue was dissolved in CH_2_Cl_2_ (100 mL). Organic layer was washed with H_2_O (2 × 50 mL) and then dried over MgSO_4_. Filtration and evaporation of the solvent yielded to compound **10** (6.9 g, 56%). R_f_ (hexane:ethylacetate 1:1) = 0.63. MS (m/z): calcd for C_23_H_22_F_13_NO_5_ 639.40 found 639.13. ^1^H NMR (CDCl_3_) δ ppm: 1.46 (m, 2H, OCH_2_C*H_2_*CH_2_CF_2_); 1.61 (m, 2H, OCH_2_CH_2_C*H_2_*CF_2_); 3.32 (t, 2H, OC*H_2_*CH_2_CH_2_CF_2_); 3.34–3.47 (m, 10H, CH_2_CH_2_O); 3.52 (t, 2H, CH_2_NPht, ^3^*J*_H-H_ = 5.76 Hz); 3.67 (t, 2H, C*H_2_*CH_2_NPht, ^3^*J*_H-H_ = 5.85 Hz); 7.48–7.55 (m, 2H, CH_Ar_); 7.57–7.63 (m, 2H, CH_Ar_). ^13^C NMR (CDCl_3_) δ ppm: 16.5 (OCH_2_*C*H_2_CH_2_CF_2_); 28.8 (OCH_2_CH_2_*C*H_2_CF_2_); 37.1 (CH_2_NPht); 67.8 (*C*H_2_CH_2_NPht); 69.3–72.0 (CH_2_CH_2_O); 72.4 (O*C*H_2_CH_2_CH_2_CF_2_); 108.1-126.3 (CF_2_); 118.5 (CF_3_); 123.1 (CH_Ar_); 132.0 (C_Ar_); 133.8 (CH_Ar_); 168.1 (C=O).

#### N-2-(2-[2-(4,4,5,5,6,6,7,7,8,8,9,9,9-tridecafluoro-nonyloxy)-ethoxy]-ethoxy)-ethylamine **(11)**

To a solution of compound **10** (6.9 g, 10.8 mmol) in absolute ethanol (120 mL) was added hydrazine hydrate (4 mL, 43.2 mmol). The solution was heated at 80 °C for 16 h. The solvent was evaporated under reduced pressure and the resulting residue was dissolved in CH_2_Cl_2_ (50 mL). After filtration the solvent was evaporated and then 5.18 g of compound was purified by chromatography (100% CH_2_Cl_2_ then 90:10 and 80:20 CH_2_Cl_2_/MeOH). Then 1.7 g of compound **11** (89%) were obtained as a colorless oil. R_f_ (dichloromethane:methanol 19:1) = 0.25. MS (m/z): calcd for C_15_H_20_F_13_NO_3_ 509.30 found 509.12. ^1^H NMR (CDCl_3_) δ ppm: 1.46 (m, 2H, OCH_2_C*H_2_*CH_2_CF_2_); 1.61 (m, 2H, OCH_2_CH_2_C*H_2_*CF_2_); 2.82 (t, 2H, CH_2_NH_2_); 3.37 (t, 2H, OC*H_2_*CH_2_CH_2_CF_2_); 3.34–3.47 (m, 10H, CH_2_CH_2_O); 3.63 (t, 2H, C*H_2_*CH_2_NH_2_). ^13^C NMR (CDCl_3_) δ ppm: 16.5 (OCH_2_*C*H_2_CH_2_CF_2_); 28.8 (OCH_2_CH_2_*C*H_2_CF_2_); 41.8 (CH_2_NH_2_); 69.3–72.0 (CH_2_CH_2_O); 72.4 (O*C*H_2_CH_2_CH_2_CF_2_); 73.2 (*C*H_2_CH_2_NH_2_); 108.1–126.3 (CF_2_); 118.5 (CF_3_).

#### 2.2.1. Protected CBO-P11 synthesis

##### Briefly, protected CBO-P11

(cyclo(D-FPQ(Trt)IMR(Pbf)IK(Boc)PH(Trt)Q(Trt)GQ(Trt)H(Trt)IGE)) was synthesized using non commercial loaded Fmoc-Glu(2-ClTrt resin)-OAll for the linear chain assembly. Subsequent Fmoc amino acids were coupled using a fourfold excess of amino acids activated as HOBt ester by means of a 0.45 M HBTU/HOBt solution. Removal of the allyl protecting group was performed before N-terminal Fmoc deprotection with Pd(PPh_3_)_4_ in a solution of CHCl_3_/AcOH/NMM (37:2:1). Fmoc removal was achieved with a solution of 20% piperidine in NMP (N-methyl-2-pyrrolidone). Coupling reaction was performed with PyBOP, HOBt and DIEA in NMP. Final cleavage of the peptide from the resin without loss of any side-chain protecting group was performed with a solution of 1% TFA in CH_2_Cl_2_. Precipitation of the product was done in cold water and filtration yielded 580 mg (65%) of protected CBO-P11 as a white solid.

##### F13-CBO-P11 **(12)**

Protected CBO-P11 (311 mg, 93 μmol) was added to a solution of compound **11** (100 mg, 196 μmol), PyBOP (242 mg, 465 μmol), HOBt (94 mg, 698 μmol), and DIEA (152 μL, 930 μmol) in 20 mL of THF. The reaction was stirred at room temperature for 48 h. Solvent was evaporated under reduced pressure; the product was precipitated in cold water and filtered. The product was then treated with 0.75 g of phenol in a TIS/thioanisole/H_2_O/TFA solution (1:2:2:40) for 3 h at room temperature. Final compound was precipitated from cold diethyl ether and filtered. The product was purified by reverse-phase HPLC under the following conditions: eluant A, 0.05% TFA in water; eluant B, 0.05% TFA in CH_3_CN/H2O (70/30); flow rate, 1 mL/min; detector 214 nm. MALDI mass spectrometry analysis (theoretical value: 2489.48 Da; experimental value: 2489.16 Da).

### 2.3. Injectable solutions

In preparation for the intravascular injection, 274 mg of FDG is dissolved in 1 mL sterile PBS. For the polyfluorinated glucose solution, 10 mg of the probe was added to 1 mL of polypropylene glycol (MW = 400 g/mol). F_13_-CBO-P11 solutions were prepared in sterile PBS. For the latter, solutions were obtained by the addition of 20 mg and 35 mg of peptide in 1 mL sterile PBS.

### 2.4. Cell culture

U87 human glioma cells (ATCC) were selected for the study as an established model for malignant gliomas that form highly vascularized tumors. Cells were maintained in culture using Dulbecco's modified Eagle's medium (DMEM) with 10% FBS, L-glutamine (2 mmol L^−1^), and antibiotics (penicillin 50 UI mL^−1^, streptomycin 50 μg mL^−1^). Cell culture was performed in a 37 °C incubator with humidified atmosphere and 5% CO_2_. Cells were grown to confluence, harvested after trypsinization, and re-suspended in serum-free DMEM for implantation.

### 2.5. In vivo injection in the chicken chorioallantoic membrane (CAM) and sample preparation

**Scheme 1.**
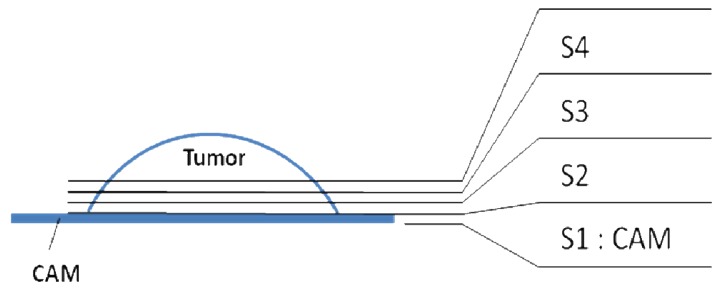
Successive sections obtained from tumor implanted on CAM.

Fertilized chicken eggs were incubated at 37 °C in a humidified atmosphere. On day 4 of incubation, a window was made in the shell in order to visualize the chorio allantoïc membrane (CAM). On embryonic day 10, in the tumor group, a ring was put on the CAM and U87 glioma cells (5 million cells per egg suspended in 20 μL of medium) were deposited on the CAM within the ring after gentle laceration of the surface. In the other group, a nylon grid was put on the CAM to allow normal tissue to grow on the grid. After 7 days of implantation (embryonic day 17), FDG, polyfluorinated glucose and F_13_-CBO-P11 solutions were then injected in a blood vessel of the CAM of each egg (100 μL per embryo). Embryos were sacrificed 6 hours after injection. All samples were removed and snap-frozen in liquid nitrogen-chilled isopentane at −160 °C. CAM samples and normal tissue (S1, Scheme 1) coming from nylon grid were then put on targets (2 mm-thick and 20 mm-diameter aluminium target with a 5 mm-hole in the centre) and freeze-dried during 3 days at−30 °C in a cryostat (CM3050S, Leica Microsystems). Tumors were conserved at −80 °C until sectioning. For tumor tissue, successive serial sections (at 100, 200 and 300 μm) were obtained from the CAM to the tumor (S2 to S4, Scheme 1) using the cryostat, put on targets and freeze-dried. After sample preparation, microphotographs were taken to monitor the experiment.

### 2.6. Ion beam microanalysis

**Figure 1.**
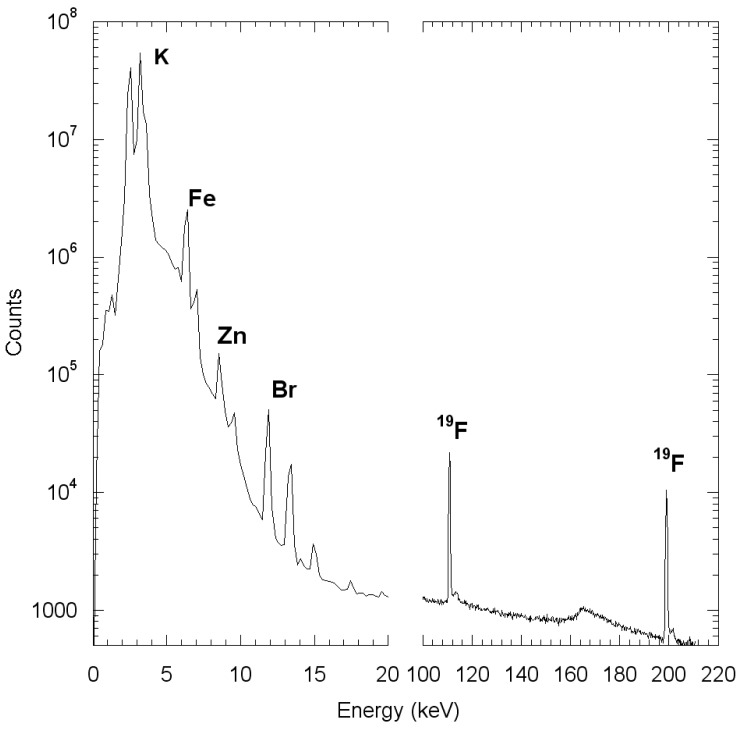
Mean PIXE/PIGE spectrum obtained on biological sample. The counts and respective energies can be sorted according to beam position in order to produce element maps showing gamma or x-ray intensity *vs.* beam position. Analytical conditions: 3.4 MeV proton beam; intensity 500 pA, beam diameter: 5 μm.

Particle Induced γ-ray Emission (PIGE) and Rutherford Backscattering Spectrometry (RBS) were performed simultaneously to determine trace elements quantitative distribution, such as F and Fe, and major element as C, N, and O determination, respectively (Figure1). Analyses were performed with proton beam energy at 3.4 MeV in order to benefit from a high cross section for the nuclear reaction for fluorine [15]. Analyses were realized on zones of typically 850 × 850 μm in a scanning mode of the proton beam. The beam was focused onto the sample surface to a spot of 5 μm in diameter, resulting in a proton beam current of 500 pA as measured with a Faraday cup below the sample. Characteristics gamma rays emitted from elements of the target were detected with an ultralow energy planar germanium detector (surface of the Ge crystal is 100 mm², thickness 10 mm) placed at 45° from incident beam. The γ-ray detector was covered by a 100 μm thick carbon filter (hole 0.2% of the total filter surface), to protect the γ-ray detector from backscattered particles. The RBS measurements were performed using a PIPS detector (passivated implanted planar silicon) placed at 135° from the incident beam direction.

CaF_2_ micromatter standard (certified material from NIST) and a PVDF film (polyvinylidene difluoride, 25 μm thick) were used as external standard in order to verify the quantification process [9]. γ-ray emission data were analyzed with the Supavisio software to create mask of interesting areas of selected chemical elements. RBS data were analyzed with SIMNRA code [13].

The quantification of fluorine was performed by comparative analysis with reference standard. PVDF films were analyzed in order to simulate the composition and thickness of biological samples. PyMCA software was used to fit the 110 keV γ peak on PIGE spectra on a PVDF film and biological samples. The intensity of the peak in the PVDF was used to normalize yield in samples [14,16].

The combination of PIGE and RBS quantitative data were used for mass normalization of y-ray emission, leading to element concentrations expressed in terms of μg of element per g of dry mass sample.

Equation of quantification can be written as follow:

$$\left[F\right]i=\left[F\right]\mathrm{Sref}\times \frac{Yi}{Y\mathrm{ref}}\times \frac{Q\mathrm{ref}}{Qi}\times \frac{1}{Mi}$$

Where: [F]i = fluorine concentration of sample i, [F]Sref = fluorine surfacic mass of the reference (PVDF, CaF_2_), Yi, Yref = intensity of 110 keV γ peak in reference and sample i, Qi, Qref = deposited charge during analysis in reference and sample i, Mi = local mass of sample i.

Detection limit of fluorine with these references was around 3 μg/g [9].

## 3. Results and Discussion

### 3.1. Synthesis of probes and precursors

**Scheme 2.**
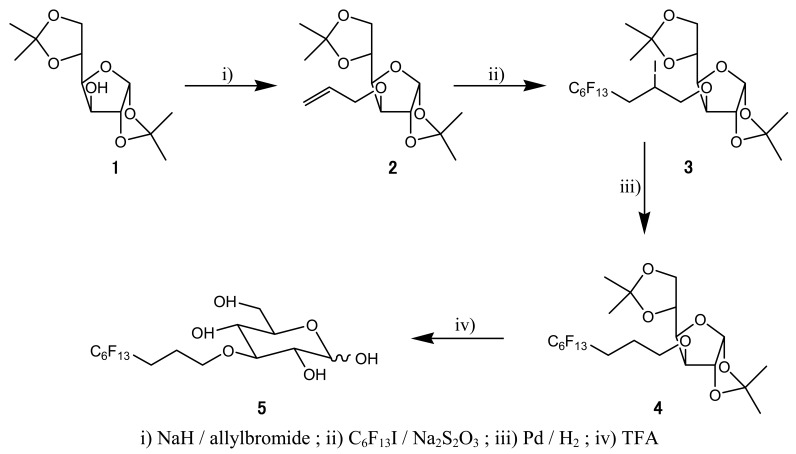
Synthesis of the polyfluorinated glucose.

The synthesis of the polyfluorinated glucose (Scheme 2) has been described by Schwabisch from 3-allyl-1,2:5,6-di-O-isopropylidene-α-D-glucofuranose **(2)** [17]. Briefly, the latter was generated by treatment of the dioxolane glucose **(1)** with allyl bromide in THF in the presence of sodium hydride. Then compound **(2)** was perfluoroalkylated by dithionite-mediated addition of the 1-iodoperfluorohexane in a solution of acetronitrile and water. After hydrodeiodination with palladium on charcoal in hydrogen atmosphere, the target product **(5)** was obtained by deprotection of the compound **(4)** with aqueous trifluoroacetic acid with a 41% overall yield.

**Scheme 3.**
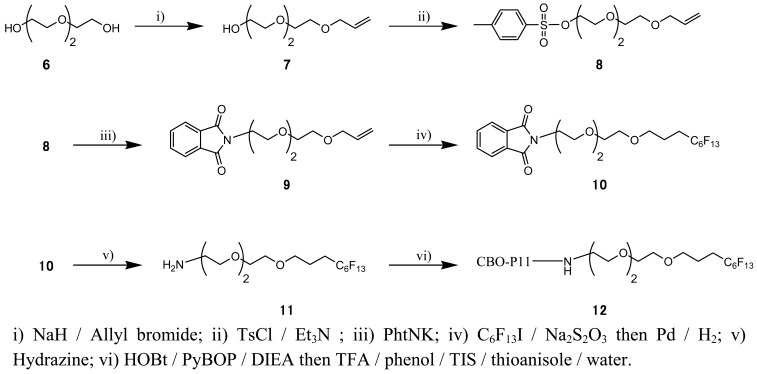
Synthesis of the polyfluorinated peptide (F_13_-CBO-P11).

F_13_-CBO-P11 (Scheme 3) was obtained by amide bond formation between polyfluorinated linker **(11)** and protected antiangiogenic peptide CBO-P11 [18], followed by a final deprotection step. Protected CBO-P11 was synthesized from 2-chlorotrityl chloride resin as previously described [18]. Polyfluorinated linker **(11)** was synthesized from triethylene glycol **(6)**. After the obtention of monoallyl derivative **(7)** (73% yield), phtalimido-allyl compound **(9)** was synthesized in two steps by treatment with tosyl chloride in triethylamine followed by potassium phtalimidate with 80% and 94% yield respectively. Polyfluorinated moiety was then introduced by dithionite-mediated addition of 1-iodoperfluorohexane followed by hydroiodation yielded compound **(10)**, which was deprotected by hydrazine treatment to give amino-polyfluorinated linker **(11)** in 27% yield.

### 3.2. Samples from FDG injected CAMs

During this experiment, two types of analyses were performed. On one hand, short analyses (2–4 h) were realized on tumor sections in order to quantify fluorine concentration. These sections were poorly vascularized and no specific accumulation of fluorine was observed. The aim of this first type of analysis was to quantify the fluorine content locally more than to image it. On the other hand, highly vascularized CAM samples were analyzed for periods of up to 22 h in order to make a map of the distribution of fluorine. The mapping of iron was also realized to confirm the presence of blood in the capillaries and help the determination of fluorine distribution. The first analysis we performed was an analysis on a CAM sample from an egg where no fluorinated tracer had been injected. This blank sample allowed us to verify that our samples did not possess naturally organic fluorine and no external contamination could distort the quantification. In addition, this test has shown that no other photon was emitted by a side reaction at 110 and 197 keV.

**Figure 2.**
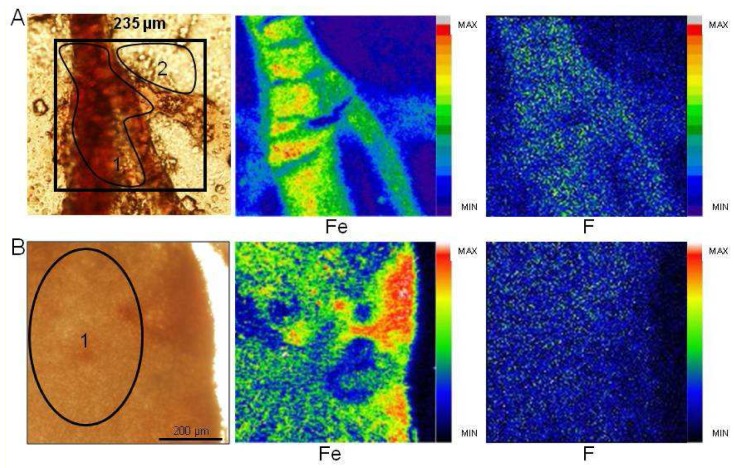
**(A)** Chemical elements distribution in CAM exposed to FDG (sample 3). Optical microscopy view of a section analyzed by ion beam microprobe (above left) and corresponding chemical element distributions maps. Average fluorine concentration: capillaries (zone 1: 1460 ± 170 μg/g), surrounding tissues (zone 2: 1820 ± 210 μg/g). (**B)** Chemical elements distribution in tumor section exposed to FDG (sample 3). Average fluorine concentration in tumoral tissue (zone 1: 1640 ± 170 μg/g). Min-max range bar units are arbitrary.

Highly concentrated solutions of FDG (0.77 and 1.53 M) corresponding to 1.46 and 2.9 mg of fluorine respectively per embryo were injected. The distribution of trace elements was first determined, and iron and fluorine maps for normal and tumor tissues were obtained (Figure 2). In the normal chorioallantoic membrane (CAM) tissue, microscopy and iron mapping allowed us to visualize well defined capillaries (Figure 2-A). Fluorine concentration was then determined from normalized samples (Table 1, samples 1 and 3), showing no significant differences in ratio between capillaries and surrounding tissues (sample 3) (1460 ± 170 μg/g dry mass of fluorine for capillaries and 1820 ± 210 μg/g dry mass of fluorine for surrounding tissue).

In tumor tissue (Figure 2-B), iron mapping indicates possibly bleeding; we then determined an average fluorine concentration in the tumor. There were no differential accumulations of fluorine between normal and pathological tissues (1820 ± 210 μg/g and 1640 ± 170 μg/g dry mass of fluorine respectively). Even at a twofold lower injected amount, the same range of fluorine distribution was obtained for sample 2 as in sample 3 (Table 1).

### 3.3. Samples from polyfluorinated glucose injected CAM

Polyfluorinated glucose, being an amphiphilic molecule, was very difficult to solubilize in PBS. Thus a 18.5 mM solution (0.46 mg of fluorine per injection) was prepared in polypropylene glycol.

Surprisingly, fluorine concentration in capillaries was found to be twice lower than surrounding tissues in both type of samples (sample 4 and 5, Table 2). Indeed, fluorine concentrations found in sample 4 (Table 2) were about 1290 ± 150 μg/g dry mass of fluorine for capillaries and 2240 ± 260 μg/g dry mass of fluorine for surrounding tissue.

**Figure 3.**
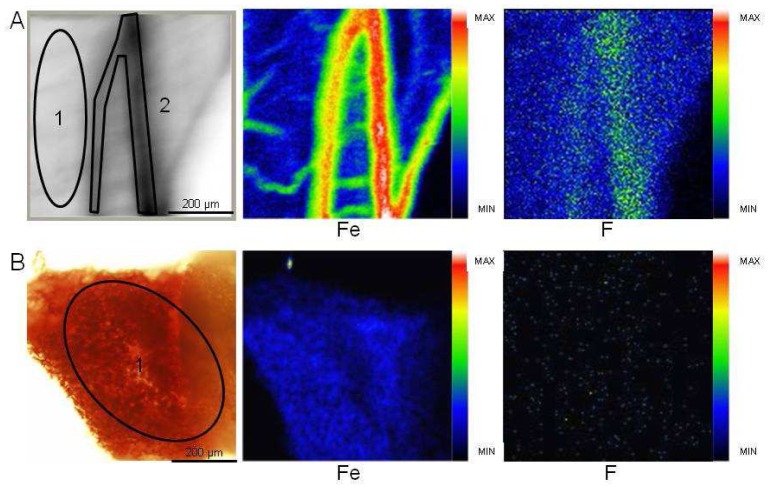
**(A)** Chemical elements distribution in CAM tissue exposed to the polyfluorinated glucose (sample 4). Average fluorine concentration: surrounding tissue (zone 1: 2240 ± 260 μg/g), capillaries (zone 2: 1290 ± 150 μg/g). (**B)** Chemical elements distribution in tumor section S4 exposed to the polyfluorinated glucose (Table 2, sample 5). Average fluorine concentration in tumoral tissue (zone 1: 130 ± 20 μg/g). Min-max range bar units are arbitrary.

The same ratio was observed for sample 5. Nevertheless, fluorine distribution has been determined (Figure 3) in accordance with capillaries identification with iron measurements. In sample 5 (Figure 3-B, Table 2), fluorine amount decreased from the CAM tissue (330 ± 60 μg/g) to the successive tumor sections (from 300 ± 30 μg/g for S2 close to CAM to 130 ± 20 μg/g for S4). The low accumulation in tumor tissue is most probably due to the fact that poorly vascularized tumors were injected (Figure 5)

### 3.4. Samples from polyfluorinated CBO-P11 injected CAM

**Figure 4.**
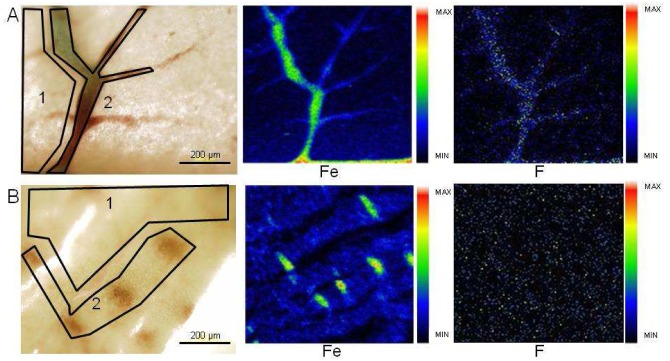
**(A)** Chemical elements distribution in CAM tissue exposed to F13-CBO-P11 (sample 8). Average fluorine concentration: surrounding tissue (zone 1: 2050 ± 175 μg/g), capillaries (zone 2: 6570 ± 555 μg/g). (**B)** Chemical elements distribution in tumor section S2 exposed to F13-CBO-P11 (sample 6). Average fluorine concentration: tumor section (zone 1: 270 ± 20 μg/g), capillaries (zone 2: 360 ± 50 μg/g). Min-max range bar units are arbitrary.

Quantification and imaging of F_13_-CBO-P11 have also been performed at two different concentrations (20 and 35 mM, which represent respectively 0.49 and 0.85 mg of fluorine injected per egg) (Figure 4).

**Figure 5.**
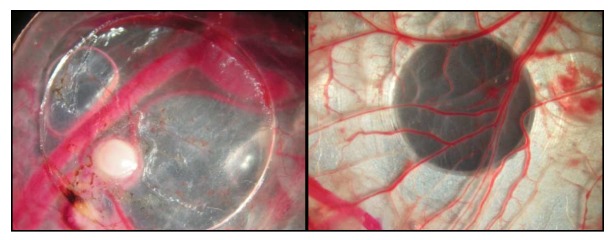
Tumor tissue with vascularisation on ring (left), CAM on aluminium target (right).

Among the studied samples, sample 6 was treated with a 20 mM solution of F_13_-CBO-P11 and samples 7 and 8 were treated with a 35 mM solution of F_13_-CBO-P11. First, we observed an incorporation of this fluorinated peptide in both normal and pathological tissues (Figure 4 and Table 3). For normal tissues (sample 8, Table 3 and Figure 4-A), fluorine concentration obtained are 3 times higher in capillaries with respect to the corresponding tissue (6570 ± 555 μg/g for capillaries and 2050 ± 175 μg/g for surrounding tissues). In the case of tumor sections (samples 6 and 7, Table 3), the fluorine concentrations in capillaries are 1.5 times higher than in the surrounding tissue (from 360 ± 50 to 650 ± 70 μg/g for capillaries *versus* 215 ± 35 to 450 ± 35 μg/g for surrounding tissues). The low accumulation in tumor tissue is most probably due to the fact that poorly vascularized tumors were injected (Figure 5).

### 3.5. Discussion

The aim of our study was to perform a first step in a multimodal multiscale imaging approach, and the second step would be an *in vivo* PET imaging of tumor angiogenic areas. An ion beam imaging analysis was designed with a higher resolution allowing the validation of biological targets at a micrometric scale. As the experimental model, we used the chicken chorioallantoic membrane (CAM). This is a highly vacularized extraembryonic tissue that has been extensively used to study vascular development [19,20] and tumor development, such as of glioblastoma xenografted onto the CAM [21-25]. We have imaged the distribution of three fluorinated compounds in the normal CAM and in the CAM xenografted with U87 glioma cells.

The first two glucose derivatives were envisaged as models to explore the limits of detection while the third could be considered as a proof of concept and a preliminary biological tool.

^18^F-FDG (2-deoxy-2-[18F]fluoro-D-glucose) is the most frequently used radiotracer for PET imaging. FDG is an analogue of glucose which compiles essential qualities that are required for a tracer. It does not perturb the measured system and follow a specific metabolic pathway. Indeed, it enters the glycolytic pathway in the same way as glucose does. After the initial phosphorylation step, the deoxy form of glucose is unable to be further metabolized and therefore is trapped within cells [6]. FDG is then concentrated in regions of higher metabolic activity, as it is known for tumors [26,27]. We therefore used FDG model as the starting point of our study.

Ion beam analyses, and particularly PIGE analysis, is several magnitudes less sensitive than PET (sensitivity of 10^−9^ and 10^−12^ mole/L respectively). Much larger amounts of glucose fluorinated tracer had to be injected to overcome this limitation. The first experiments were performed with highly concentrated FDG solutions (0.77 and 1.53 M). This concentration is much higher than physiological glucose concentrations in the blood (7.2 mM). As the total blood volume of a 17-day chick embryo is about 2 mL, injection of 100 μL of these solutions increases blood glucose from 7.2 to 45.7 mM and 83.7 mM respectively. Our results demonstrate a homogeneous fluorine distribution within capillaries as well as in surrounding tissues (about 1600 μg/g average concentration) (Table 1). The concentrations of fluorine detected in sample 2 and 3 were close to each other. However the injected amount is twice less in the case of sample 2 than in sample 3. This may be due to the high FDG diffusion due to tissue homeostasis. These results demonstrate that fluorine can be detected using ion beam analysis. In any case, the ratio of fluorine concentrations between capillaries and tissues is close to one.

We next synthesized polyfluorinated tracers to increase the amount of fluorine injected into embryos necessary for PIGE detection, the first one being a glucose derivative. Previous studies on chemical modifications of glucose have been carried out in order to determine which glucose substitution was the most suitable. Positions 2 and 3 seem not to significantly change glucose internalization and phosphorylation in cells [28,29]. We therefore decided to insert a polyfluorinated chain on glucose position 3 (Scheme 2). Even at an amount of fluorine injected per embryo about 3 times lower than that in samples incubated with 0.77 M FDG solution (0.46 mg of fluorine compared to 1.46 mg) and more than 6 times for 1.5 M FDG solution (2.9 mg of fluorine), fluorine maps (Figure 3) and local mass (Table 2) distribution could be obtained. In normal CAM tissue, we obtained about 2.5 times lower concentration in capillaries than in surrounding tissues.

To improve vascular targeting and to have in hand a potential proof of concept for imaging of angiogenesis, we used CBO-P11 (cVEGI), a peptide which binds receptors involved in angiogenesis. It has been shown that CBO-P11 inhibits angiogenesis and tumor development *in vivo* in the CAM assay and in animal experiments using rodents [30]. CBO-P11 was first functionalized as F_13_-CBO-P11 (Scheme 3) in order to enable its detection by PIGE. As previously demonstrated [18], the addition of chemical groups on CBO-P11 does not change the affinity of the peptide to its binding site. Chicken embryos were then injected with F_13_-CBO-P11 (Figure 4 and Table 3) and fluorine concentrations/amounts measured were 3 and 1.5 times lower in surrounding tissues than in capillaries in normal and tumor CAM tissue respectively. The fact that similar fluorine concentrations were detected in sample 6 and 7 demonstrate that fluorine uptake is not dose dependant at the amounts used in our study.

It is important to notice that the low absolute concentration values in tumor tissue are due to the fact that the injected tumors were poorly vascularized. However, in this “prevascular” state, we may hypothesize a high expression level for growth factors receptors. CBO-P11 exhibit a high affinity for these, which could explain the relative higher fluorine concentration in tumor *vs.* capillaries than in normal tissue *vs.* capillaries.

## 4. Conclusions

This article describes for the first time the detection of organic fluorine in biological samples with the use of PIGE. This technique was successfully applied to quantify fluorine in chorioallantoic membrane (CAM) tissue and in tumors xenografted on the CAM. It moreover allows to estimate the ratio of fluorine concentration between capillaries and surrounding tissues, either normal or tumors. These results demonstrated that this technique, using the ^19^F(p,p′γ)^19^F reaction, is an accurate technique for fluorine analysis and presents good sensitivity with a LOD in the low ppm range.

After validation of the experimental model using fluorinated glucose derivatives, CBO-P11 (cVEGI), an antiangiogenic peptide was functionalized with a polyfluorinated linker and used for PIGE analysis.

PIGE analysis is a new technique which constitutes the first step of multimodal multiscale imaging. It allows analyzing fluorine distribution in biological tissues with a precision enabling *ex vivo* assessment in order that tracers effectively hit biological targets for which they are designed. This will allow *ex vivo* imaging of labeled molecular probes and validation of their biological targets at a higher resolution than PET, *i.e.,* cell scale. The second step will use PET imaging which will give *in vivo* information. The previously mentioned PIGE analysis will compensate the lack of precision. Both approaches used complementarily will increase the quality of data generation for medical cancer imaging.

## Figures and Tables

**Table 1.** Fluorine concentration in normal and tumor tissues exposed to FDG solutions (0.77 and 1.53 M).

**Sample**		**Average fluorine concentration (μg/g)**		

		**Physiological tissue**		**Pathological tissue**
**Tissue (injected solution concentration)**	**Successive sections**	**CAM**	**Capillaries**	**Tumor**
Sample 1 (1.53 M)	S1	1560±180	960±100	-

Sample 2 (0.77 M)	S1	-	-	1570±180

Sample 3 (1.53 M)	S1	1820±210	1460±170	-
	S2	-	-	1640±170
	S3	-	-	1950±240
	S4	-	-	1470±140

Sample 1 (S1): CAM sample (no tumor); Sample 2 (S1): tumor section (no normal tissue); Sample 3 (S1): CAM sample (no tumor); Sample 3 (S2 to S4): serial tumor sections (no normal tissue). Refer to Scheme 1 and the text for more details.

**Table 2.** Fluorine concentrations in normal or tumor tissues exposed to polyfluorinated glucose (18.5 mM).

**Sample**		**Average fluorine concentration (μg/g)**		

		**Physiological tissue**		**Pathological tissue**
**Tissue (injected solution concentration)**	**Successive sections**	**CAM**	**Capillaries**	**Tumor**
Sample 4 (18.5 mM)	S1	2240±260	1290±150	-

Sample 5 (18.5 mM)	S1	330±60	95±20	-
	S2	-	-	300±30
	S3	-	-	140±30
	S4	-	-	130±20

Sample 4 (S1): CAM sample (no tumor); Sample 5 (S1): CAM sample (no tumor); Sample 5 (S2 to S4): serial tumor sections (no normal tissue). Refer to Scheme 1 and the text for more details.

**Table 3.** Fluorine concentrations in normal and tumor tissues exposed to fluorinated CBO-P11 solutions (20 and 35 mM).

**Sample**			**Average fluorine concentration (μg/g)**			

			**Physiological tissue**		**Pathological tissue**
**Tissue (injected solution concentration)**		**Successive sections**	**CAM**	**Capillaries**	**Tumor**
Sample 6 (20mM)	S1	-	-	450±35	610±70
	S2	-	-	270±20	360±50

Sample 7 (35 mM)	S1	-	-	345±30	650±70
	S2	-	-	215±35	460±45

Sample 8 (35 mM)	S1	2050±175	6570±555	-	-

Sample 6 (S1 and S2): serial tumor sections (no normal tissue); Sample 7 (S1 and S2): serial tumor sections (no normal tissue); Sample 8 (S1): CAM sample (no tumor). Refer to Scheme 1 and the text for more details.
